# "The Hospital" Nursing Mirror

**Published:** 1901-09-14

**Authors:** 


"The Hospital" Nursing Mirror.

Being the Nursing Section of "The Hospital."

[Contributions for this Section of "The Hospital" should be addressed to the Editor, "The Hospital" Nursing Mirror, 28 & 29 Southampton Street Strand, London, W.C.]

### Notes on News from the Nursing World.

### NURSING PRESIDENT McKINLEY.

It is satisfactory to know that, if the precious life of the President of the United States can be saved by means of medical skill and good nursing, the assassin will have fired his bullets in vain. The President, after the attempted murder, was taken to the hospital in the grounds and placed upon the operating table. At the conclusion of the operation he was at once conveyed to the residence of Mr. Milburn, close to the Exposition. Mrs. McKinley, who is often with her husband, has lately shown her high appreciation of the value of careful nursing. To Miss Hunt and Miss Taylor, the two nurses who attended her with such devotion during her serious illness, she presented a valuable gold ring in token of her regard. From the outset two trained nurses have been in constant attendance upon the President, as well as three orderlies, and on Tuesday a third, Miss Grace McKenzie, was summoned to Buffalo. In the most favourable circumstances their services will be required for a considerable time to come, and we can only hope that they will be afforded the joy of witnessing the entire recovery of the distinguished patient.

### MENTIONED IN THE DESPATCH.

The final despatch of Lord Roberts in his capacity of Commander-in-Chief of the British forces in South Africa is of great interest to nurses, for it contains the names of a number of ladies whose special and meritorious services he desires to bring before the notice of the Secretary for War. First come the following members of the Army Nursing Service:—Superintendents Miss M. Thomas, Miss S. J. Browne, Miss L. Hardement, Miss E. A. Dowse, Miss S. E. Webb, Miss S. E. Oram, Miss A. Garriock; Nursing Sisters and Acting Superintendents Miss L. W. Tolloh, Miss L. M. Stewart; Nursing Sisters Miss E. T. Noble, Miss A. S. Bond, Miss J. Hoadley, Miss M. G. Hill, Miss A. A. Murphy, Miss H. L. Neale, Miss A. R. Rose-Innes, Miss L. M. Culverwell. Next are the members of the Army Nursing Service Reserve, namely:—Nursing Sisters E. A. Chaffey, A. Knaggs, F. Holmes, E. E. Coutts, C. M. Friend, M. L. Harris, E. McC. Anderson, E. A. Deacon, L. Warriner, T. Davis, F. C. A. Holcroft, G. Balfour, M. O'C. McCreery, E. A. Snape, A. Beadsmore Smith, J. E. Skillman, M. I. Burdett, M. F. Lightfoot, A. E. Davidson, L. B. Peers, E. H. Becher, C. S. McGowan, Mrs. E. K. Hamilton, A. B. Trew, E. M. McCarthy, M. E. Greenham, J. Southwell. Under the head of the "Civil Staff, Kimberley," are the following:—Miss Gordon, matron, Kimberley Hospital; Miss Bainbridge, sister; Miss Childs, sister; Miss Couch, sister; Miss Jewel, sister; Miss Nicolson, sister; Miss Strickland, sister; Miss Wallace, sister; Sister Henrietta, St. Michael's Nursing Home; Nurse Watkins, Miss F. Brice, Mrs. Ashe, Mrs. Cornwall, Mrs. Watkins. And under that of "Civil Staff, Mafeking":—Lady Sarah Wilson, matron, Convalescent Hospital; Mother Superior Teresa, Sister of Mercy; Miss Craufurd, matron, Children's Hospital; Miss C. Hill, matron, Victoria Hospital. The names of Miss D. Nealy, Miss E. R. Turner, Miss Julia Underwood, Mrs. Eugenie Ludlow and Miss Gertrude Kingston are also mentioned in the despatch. Finally, Lord Roberts having "once more" drawn attention to the great civil hospitals in South Africa, "which did so much to alleviate suffering and to moderate the strain thrown on the Royal Army Medical Corps," recites the names of persons who helped to raise and equip them, and to maintain them in a state of efficiency, including Miss MacDonnell, Nursing Sisters Walker, Denton, Smyth, McGonigal, and Richardson, of the Irish Hospital; Countess Howe, Lady Chesham, Matrons Nisbet and Fisher, of the Yeomanry Hospital; Viscountess Parker, Miss Marion Lloyd, of the Army Nursing Service Reserve, of the Welsh Hospital; Sister Ella Lawrence, of Princess Christian's Hospital; Sister A. W. Gill, of the Edinburgh Hospital; Matron E. C. Shannon, of the Scottish National Hospital; and Nurses Edith Pretty, Frances Russell, Alice Maud Davies, and R. A. C. Davies, of the Portland Hospital.

### THE FUND FOR SICK WAR NURSES.

An appeal has been issued by Georgina Lady Dudley, which merits the warmest support of all sections of the community. Twenty-one months ago Lady Dudley started a fund for the assistance of sick officers and nurses, and she has been able, by the liberality of friends and others, to expend £2,258 of it for the benefit of 217 nurses. About 50 per cent. of these have been connected with the Army and the Army Nursing Reserve. The remainder were colonial and volunteer nurses, in charge of sick and wounded on the home-coming transports, many of whom were themselves invalids greatly in want of rest. A few have had to undergo operations. The need for hospitality in the case of the colonial and volunteer nurses is very urgent, because their pay ceases on the date of disembarkation, and every transport which arrives adds to the number of them. Lady Dudley's appeal for contributions is warmly endorsed by the Lord Mayor of London, and by the Central British Red Cross Committee. The latter emphasises the importance of the organisation which she established for dealing with distressing cases of serious illness and grave injuries among officers and nurses sent invalided home from the war, and the Lord Mayor says that the resources of the Mansion House and Lloyd's Patriotic Funds which have helped her with grants are now exhausted, and that therefore she is compelled to beg the public to enable her to continue her good work. Lady Dudley herself observes that "In future emergencies all this will be taken over by the Government," and, of course, the duty belongs to Government who have shirked it so far as the South African campaign is concerned. The public, we hope, will take care that Lady Dudley is placed in a position to make up for their shortcomings.

### IRISH GUARDIANS AND THE LOCAL GOVERNMENT BOARD.

A Dublin correspondent, who sends us a brief recapitulation of the facts in connection with the question of nursing in Irish workhouse infirmaries, necessarily traverses some of the ground we have already covered. But our correspondent's *resumé* deals, in addition, with some of the statements made at the recent conference, and in particular with that of the chairman, Mr. Joseph Mooney, who in his anxiety to discount the value of trained nurses, declared that with sufficient money a nurse could get a certificate in some of the recognised hospitals in twelve months, while another speaker asserted that, under certain conditions, it could be obtained in three. Our correspondent, by a citation from Article II. of the Order of July 5th, shows that the Local Government Board will only recognise the term "trained nurse" as applying to any person who has resided "for not less than two years in a general, clinical, or other hospital recognised by us, and who, after examination, has obtained from such hospital a certificate of proficiency in nursing." Another allegation at the conference was that high salaries had to be paid to trained nurses owing to the monopoly held by a few hospitals in Dublin. This also is entirely disposed of by our correspondent, who, however, adds that the result of the conference is likely to be an attempt to induce Mr. Wyndham, or the Local Government Board, to modify the Order. We trust that no suggestion of the kind will be entertained. The conditions laid down in the Order are moderate in the extreme. In due time, we hope that the qualification which is now fixed at two years will be raised to three. It is not an unfavourable sign that only 20 out of 100 unions in Ireland were represented at the Conference.

### ONE PROBATIONER FOR SIXTY PATIENTS.

The Walsall Board of Guardians are guilty of worse offences than that of allowing the master of the workhouse to affront the nurses by ordering the domestic servants to wear similar uniform. They have ignored recommendations of the Local Government Board Inspector, and they have permitted a condition of things in the workhouse infirmary which can only be described as scandalous. The medical officer, Dr. Fox, stated in his report, which was read at a meeting of the Guardians, that the nursing staff was quite inadequate, and "that only recently, at night, he had been compelled to call up a nurse who had been on duty all day." On the same occasion a letter was read from the Rev. E. C. Delaney, Roman Catholic priest, stating that he considered the nursing arrangements at the infirmary little less than a scandal. "The other night he was summoned to a sick bed, and noticed that one probationary nurse had sole charge of two wards, in which were sixty patients, some of the cases being most serious and requiring the almost constant attendance of a nurse. Three patients, who were delirious, persisted in getting out of bed whenever the nurse's back was turned, and it was a marvel to him that one of them did not meet with a serious accident by falling downstairs." After this evidence the chairman was compelled to recognise the necessity of an increase in the nursing staff, and the medical officer was empowered to engage "an additional nurse." He was also asked to report to the Guardians what further increase was necessary. It is very clear that considerably more assistance than that of one additional nurse is obviously imperative, both in the interests of the neglected patients and in those of the overworked nurses, but in any event we think that it is very doubtful whether there will be any disposition on the part of competent nurses to serve under Guardians who have done so much to cause their training school to be shunned.

### A CRY FROM ASTON.

Bad as the condition of affairs is at Walsall Workhouse Infirmary, it appears that, in one sense at all events, things are worse at Aston Infirmary. The correspondent of a Birmingham paper states that at the latter institution one nurse has over 80 patients, and for a portion of her time 150 inmates, to attend to. The greater number of the patients, it is added, are bed-ridden. At Aston, moreover, there is said to be no paucity of nurses, in which case the responsibilities imposed upon a single nurse are the more indefensible. Have the Guardians gone to sleep?

### THE TRAINING AT AN OPEN-AIR HOSPITAL.

In an interview between the lady superintendent of the North London Consumption Hospital and our commissioner, which appears in another column, the duties of the nurses in an institution where the open-air treatment has been in full swing for some years are described in detail. The lady superintendent lays stress on the fact that probationers are received at the age of 21. As the period of training is for two years, this affords a young woman who has determined to pursue a nursing career an opportunity of obtaining valuable experience before she enters a general hospital. The new sanatorium in Hertfordshire, in connection with the hospital at Hampstead, will soon be in course of erection, and a considerable staff of nurses will be required as soon as it is completed. Some of the numerous girls who write to us expressing anxiety to enter a training school, but have not reached the minimum age of admission to a general hospital, may thus find the opening they desire.

### THE PRELIMINARY TRAINING HOME AT THE "LONDON."

An important weekly paper has just published some particulars of the preliminary training for probationers at the London Hospital. They are, we understand, entirely correct. But they scarcely possess the merit of novelty. The Preliminary Training Home at the London was opened in June 1895. It has worked admirably ever since, and is likely to continue on the same lines. There have been no alterations in the regulations.

### THE SITUATION AT BELFAST.

Following the serious statement of Dr. Ritchie that the nurses in the Belfast Workhouse Infirmary are "living in a culture bed of typhoid fever," Dr. Robb, the medical officer of the hospital, has urged upon the Board of Guardians the advisability of making permanent provision for the housing of the nursing staff outside the hospital. In the report containing this recommendation Dr. Robb says: "The accommodation for the nurses in the hospital is altogether unsuitable and insufficient, and the taking up of many of the wards intended for the reception of patients seriously interferes with the management of the hospital. This is especially felt during epidemics such as the present." According to the chairman, everything will be put right when the new nurses' home is ready, but meanwhile, we suppose, the unfortunate nurses must continue to reside in "a culture bed of typhoid."

### A SCARCITY OF SISTERS.

We learn with some surprise that an advertisement for a sister for enteric wards at the Fever Hospital, Leeds, did not produce a single suitable application. There are 32 beds, salary is £32 increasing to £36, and indoor uniform is provided. Three years' general and fever training are indispensable. The Leeds Fever Hospital enjoys an excellent reputation, and we should have thought that there would have been no difficulty in securing the services of a fully-trained nurse for the post of sister.

### AN AVOIDABLE CALAMITY.

At the annual meeting of the supporters of the Chesterfield Victoria Home for Nurses, the Rev. E. G. Stukeley, in moving the adoption of the report, testified to the value of the district nurse in the poor parts of the town, and said that he felt "it would be nothing short of a calamity if the home were closed for want of the necessary funds." It is, however, impossible to believe that the Chesterfield people will allow a calamity which is so easily avoidable to occur. Last year's report is certainly not satisfactory, as the committee of the home had to fall back on their reserve fund in order to keep things going, and reserve funds when encroached upon for the purpose of paying ordinary expenses, have a habit of melting away very quickly. We note that one of the speakers affirmed that the work of the district nurse constitutes a main claim of the home to public support. This is true, and we hope that there is no risk of the district work being crippled by the needs of the paying branch.

### THE PROSECUTION OF A NURSING INSTITUTE.

We are frequently asked by nurses of both sexes to state the conditions upon which they would be allowed to start a home for the treatment of mental cases; and our invariable reply is not to attempt anything of the kind without the sanction of the Lunacy Commissioners. The conviction of the proprietors of a Medical and Surgical Home and Nursing Institute at Kew for receiving and taking charge of lunatics in an uncertified institution is, however, an object lesson which will doubtless carry more weight than any general advice. This was a case in which there was no suggestion of improper treatment. Moreover, the defendants averred, honestly enough, that they believed the patients were not insane, but only paralysed. On the other hand, it was urged, at the instance of the Lunacy Commissioners, that the belief that the patients were sane was not material to the case; and though three local practitioners said that they did not consider them to be "certifiably insane," the Richmond bench imposed fines and costs amounting to nearly £60. In the circumstances, the punishment seems unduly heavy, and as the defendants are well known in Richmond, it savours of unnecessary harshness on the part of the magistrates to have refused to afford them reasonable time for payment.

### DISTRICT NURSES AT WOOLWICH.

The annual report of the Woolwich, Plumstead, and Charlton Nursing Association for 1900 surveys the work of the nurses during the past ten years, during which period it has increased enormously—starting in 1891 with one nurse and £103 to spend, while in 1900 six nurses were at work and the amount raised was £501. The total number of visits paid during the year was 13,749, exclusive of 311 visits to the *Warspite* training ship, at each of which the nurse attended an average of 20 boys. This extension of the work took place early in the year when, at the suggestion of the medical officer, Dr. C. T. W. Hirsh, an arrangement was entered into by the committee by which the association undertakes the nursing of the boys and receives payment in proportion to the time taken up. A nurse attends at a fixed hour every morning to carry out the doctor's instructions, and an hour or so is spent on board ship mainly on minor ailments and dressings. The nurses also inspect all the Country Holiday Fund children from the district—a work which involves a good many hours and much judgment and tact. Several lectures on nursing were given during the year by the superintendent, Miss Goodwin.

### THE MEMORIAL TO MISS HANNAN.

Many of our readers will be interested to know that the grave and memorial cross to the late Miss Hannan, who was for so many years the esteemed lady superintendent of Crumpsall Infirmary, are now completed, and may be inspected at the Greenbank Cemetery, Bristol. Those who are unable to visit the city can obtain photographs of the grave and cross from Mr. Spencer, 20 Berwick Road, Stapleton, Bristol.

### LODGING-OUT AT PLYMOUTH.

It appears that of the staff of sixteen nurses at the Plymouth Workhouse Infirmary, only five are provided with lodgings in the institution. The cost of those who are compelled, under existing circumstances, to lodge outside is 64s. per week. At a meeting of the Board of Guardians the other day, this system, or want of system, was discussed at some length, and with considerable animation. The proper remedy is the erection of a nurses' home, and we are glad to hear that a move in that direction is seriously contemplated. Complaints were made that the number of nurses at Plymouth exceeds the regulations of the Local Government Board, but it transpired that there have only been two more to take the places of those who are away on holiday, and that two are leaving shortly.

### SHORT ITEMS.

The Victoria Scholarship Fund, initiated by Lady Curzon and intended to give proper training to women in midwifery and nursing is receiving a satisfactory amount of support. The ninth list of subscriptions shows a total collection of upwards of £30,000 contributed to this excellent movement for recognising the abiding interest taken by Queen Victoria in the welfare of the women of India.—Nursing Sisters L. M. Monk and J. E. Garner were discharged from hospital to duty in the week ending September 1st, and Nursing Sisters C. Brown, R. M. Bullock, and F. Low have been invalided home from South Africa. The latter are on board the *Tagus*, which left Capetown on August 31st. Nursing Sisters B. Brooks, C. Terry, E. Fry, and M. A. Franklin are on passage home in the same vessel. The s.s. *Avoca* has again arrived at Southampton, the following nursing sisters forming the ship's staff: M. A. Makepeace (superintendent), J. Ward, A. Cullam, B. Milne, A. Smith, M. Estcourt, and A. E. Byrne. Sister Milne is time expired, the rest rejoin the ship.—A Queen's Nurse has been engaged by the March Nursing Association, and entered upon her duties last week. Upwards of £85 has now been subscribed as the result of the committee's appeal.—Among other benefactions Mr. Andrew Barlow, of Shirley, Southampton, has given the sum of £3,250 to the Southampton Branch of the Queen Victoria's Jubilee Nurses' Institute.—By permission of the Prime Minister, a meeting was held at 20 Arlington Street, on Tuesday, in order to complete the organisation of the Women's Memorial to Queen Victoria for the county of Hertfordshire. The Countess of Lytton having expressed her pleasure in being able to interest the county in a memorial to Queen Victoria of so appropriate a nature, Lord William Cecil appealed to those present to second Lady Lytton's efforts. It was finally resolved to canvass the county in detail, in order that everyone might have an opportunity of contributing to the scheme.

### Lectures to Nurses on Medicine and Medical Nursing.

By NORMAN DALTON, M.D. London, F.R.C.P., Physician to King's College Hospital.

### LECTURE XIX.—ALBUMINURIA—BRIGHT'S DISEASE —URÆMIA.

ALBUMEN occurs in the urine under various circumstances, such as congestion of the kidneys in mitral disease, inflammation of the kidneys, etc., and it was the work of Dr. Bright to point out that certain special cases of albuminuria were due to definite changes throughout the tissues of both kidneys. The name of Bright's disease has, therefore, been given to these special cases, and they are divided into four varieties. The first is "acute nephritis," such as occurs after scarlet fever, or after exposure to cold. This is often called acute Bright's disease. It must be understood that these are special kinds of inflammation, and that those cases of inflammation in which pus is poured out and abscesses form in the kidney are not cases of Bright's disease. The other three varieties are all called chronic Bright's disease, and they comprise the "large white kidney," the "small granular kidney" and the "lardaceous kidney." The first two names are derived from the appearance which the kidney presents on the post-mortem table, and the third is so called because a substance called lardacein is found in the organ. Lardaceous disease of the kidney (and also of the liver, spleen and intestines) occurs in persons who have suffered for a long time from suppuration (such as is due to tubercular disease of bones, or phthisis, etc.). It also occurs in cases of syphilis. The important thing for a nurse to remember is this: that the earliest sign of a lardaceous kidney is a large increase in the amount of urine passed per day; and this circumstance, if noted in the course of a case of spinal or hip disease, must be reported at once.

The physician diagnoses a case of Bright's disease by the examination of the urine. He also obtains valuable information by examining the heart, the arteries, the retina, and the skin (for dropsy). The symptoms mentioned by the patient are also important, and it may be as well here to correct the popular error that pain in the back is a sign of Bright's disease. In the acute form there may be slight aching across the loins, but the chronic forms are painless. Now, putting aside the examination of the urine, and taking all cases of Bright's Disease together, it is possible to divide all the symptoms and signs into two groups. The first group comprises those symptoms which are due, more or less, to the loss of albumen, and the second group those symptoms which are due to a poison in the blood. The latter are called "uræmic" symptoms.

The symptoms produced by the loss of albumen are "weakness" and "dropsy." Albumen exists in great quantity in the serum of the blood, and forms the chief nourishing quality of that fluid. Now, as nearly all the albumen which is found in the urine in Bright's disease has been drained directly from the blood into the renal tubes (where it mixes with the urine), it follows that, whenever there is a large quantity of albuminuria, the blood is deprived of a great deal of its nourishing quality, and consequently the patient becomes extremely weak. If it be considered that in a bad case an ounce of urine may yield after boiling three-quarters of its bulk of solid albumen, it is obvious that if 30 or 40 ozs. of such urine be passed every day, the total loss of albumen to the blood will be very large indeed. As a result the patient becomes incapable of muscular or even mental work, and presents all the symptoms of extreme "asthenia" (see Lecture II.). Dropsy is also due indirectly to the loss of albumen, but this symptom has been fully discussed in Lecture VI. With the dropsy there are associated a pallid complexion and an apathetic look. As a rule when the urine contains a great deal of albumen the quantity passed per diem is smaller than normal, so that a good deal of water is retained in the body, which doubtless helps to cause the dropsy.

In some cases of Bright's disease the blood becomes saturated with a poison which produces a number of serious symptoms. This condition is called "uræmia." We do not know exactly what the poison is. It was at first thought to be "urea," because it seemed natural to suppose that when the kidney was diseased it would no longer remove the urea from the blood, and that consequently the urea would remain in the blood and act as a poison. But it is now certain that the poison in uræmia is not urea. The symptoms of uræmia are: (1) Persistent "headache" of a dull aching character, mostly situated in the forehead, most frequently present in the morning but often coming on suddenly at any time of the day. (2) Peculiar uncomfortable sensations in the legs or actual "cramp" in the calf muscles. (3) Attacks of "vomiting," not produced by errors of diet and not attended by pain in the stomach, but rather by giddiness. (4) "Dyspnœa." This is called renal asthma. The breathing may be loud and wheezing as in ordinary asthma, but in many cases the air can be heard entering the chest in a perfectly healthy manner, although the patient is breathing very rapidly and is greatly distressed. The cause of the dyspnœa is not that the air cannot reach the blood in the lungs, but that the poisoned blood cannot take up oxygen. A short dry cough may accompany the dyspnœa. (5) "Hæmorrhages." In bad cases of uræmia small hæmorrhages may occur in the skin, especially on the legs. They are red at first, but soon become dark purple, and they do not disappear when the skin over them is pressed by the finger. They are called "petechiæ" or "purpuric spots." In some cases a large quantity of blood is vomited, or passed from the bowel or expectorated. Hæmorrhages are bad signs in all cases of kidney disease. 6. "Convulsions." These are common in cases of uræmia. Sometimes they come on suddenly in a patient who did not appear to be seriously ill. They may resemble an epileptic fit, *i.e.*, the patient may become suddenly unconscious with rigidity of all the muscles, so that the body is greatly contorted. Even the diaphragm is rigid, and, as the patient cannot breathe, his face becomes blue. This is the first, or "tonic," stage of the convulsion, and it only lasts a few seconds, for otherwise the patient would die of arrest of respiration. It is immediately followed by the second or "clonic" stage, during which the limbs are jerked about, and the facial muscles work so as to produce horrible grimaces. The tongue is frequently bitten during the fit. This stage lasts several minutes, and in uræmic convulsions as distinguished from true epilepsy, the patient may pass rapidly from one fit into another, and the two stages may not be clearly divided one from the other, the clonic stage predominating. In other cases uræmic convulsions come on gradually. There may be a slight flicker of an eyelid, a twitch of one corner of the mouth, or sudden jerky movements of the fingers. These signs are of immense importance as they may be followed at any moment by a general convulsion. It may be mentioned here that the convulsions which occur in lying-in women are nearly always due to uræmia and have the characters just described. (7) "Coma." This word means a state of unconsciousness, and, in uræmia, unconsciousness may set in without any convulsions. As a rule, it comes on gradually. The patient becomes drowsy and perhaps giddy. He doses and mutters in his sleep. If roused he may be incoherent or delirious, and eventually he becomes so unconscious that he cannot be roused at all. The pupils are equal and usually contracted, the breath is foul and ammoniacal in odour, and the tongue is dry and brown. The urine may be extremely scanty, but is not necessarily so. This state of coma very often ends in death.

### The Nurses of the North London Consumption Hospital.

(By Our Commissioner.)

A CHAT WITH THE LADY SUPERINTENDENT.

One of the first questions I asked the Lady Superintendent of the North London Consumption Hospital, after I had been over the wards, and seen the latest improvements in the well-known institution on the heights of Hampstead, was about the new Sanatorium which it is intended to erect in Hertfordshire on a site acquired for the purpose.

"Building will be commenced as soon as possible," said Miss Whitton, "but though the ground has been cleared, the plans are not yet out. I can, however, tell you that there will be accommodation for 130 patients, and that the nursing staff will probably consist of a matron, an assistant matron, and about 20 nurses. There will be a laundry large enough to do the washing for 300 persons, with eight laundresses always at work.

A Large Laundry.

"Then you will send the linen from here to Hertfordshire?"

"Yes; now it has all to be put out. The disinfector which you saw just now was put up a couple of months ago at a cost of over £300. The whole of the linen is disinfected before it is sent to the wash, and aired when it is returned. But it will be a great advantage to have our own laundry."

"Will patients also be sent from Hampstead to the new sanatorium?"

"A certain number I expect, but some may be sent from the central out-patient department in Fitzroy Square, and others will go direct. I am not quite sure, however, what will be done."

The Introduction of Open-Air Treatment.

"This was the first hospital in London to start the open-air treatment?"

"Yes; we began with two little balconies. At that period we had only 60 patients and only seven nurses. Now there are 100 patients, of whom 55 are men and 45 women, and 16 nurses, of whom three are sisters. I should prefer 17, in order to have an extra probationer available in case of need. The introduction of the open-air treatment, three years ago, rendered it necessary to alter the entire system. I have been lady superintendent from the time the change was made. Previously, I was sister in charge of the Friedenheim Home for the Dying for five years. I was trained for three years at 'Her Majesty's Hospital,' Stepney Causeway, and was charge nurse there until I went to Swiss Cottage. I have spent a few days at Nordrach, and seen as much as I could of the German method."

The Staff and Hours.

"What are the duties of the sisters?"

"One is in charge of the men's corridor and another of the women's corridor during the day, while the third is in charge of all the wards at night. All the sisters have been trained three years. The day sister in the men's wards has five probationers and a staff nurse under her, and the day sister in the women's wards three probationers and one staff nurse. During the night there are on duty, in addition to the sister, one staff nurse and two probationers."

"And the hours on duty?"

"The probationers come on duty at 8 and go off at 9. They have two and a half hours off each day, and on Sunday the afternoon or evening alternately, also a whole day once a month. I try to give them a fortnight's holiday at the end of each six months, so that they may get away from the atmosphere as frequently as possible. The night sister comes on duty at 9, and has some hours off one evening every week. She has a fortnight's holiday at the end of each three months."

The Night Sister.

"I suppose her work is especially trying?"

"It is very wearying. She has to be constantly going round the wards all through the night. The night sister receives from the doctor instructions concerning the patients as to which class they are to be placed under."

"How many classes are there?"

"Five. 'A' patients are in bed all day, 'B' get up for a couple of hours, 'C' for a little longer, 'D' can go in the grounds, 'E' patients are allowed to be on the heath for an hour. Of course it depends upon the temperature and the general condition of the patients to which class they are assigned. All patients but those suffering from the heart or from bronchitis are treated in the open air."

Probationers' Training.

"What is the minimum age at which probationers can enter?"

"Twenty-one. Sometimes we have probationers as old as 30, but I prefer the age to be one or two and twenty. The period of training is two years. Staff nurses must be here for a year. They should have had previous training at some other hospital. Afterwards I always recommend both staff nurses and probationers to move on to a general hospital when they leave us. Charing Cross Hospital will receive any probationers recommended by me. It is an obvious advantage for girls of twenty-one or twenty-two to come to us, because they cannot at that age enter a general hospital, but I invariably advise applicants of twenty-four or twenty-five to seek admission to a general hospital at once."

The Supply.

"Do you get plenty of applications?"

"More than enough of a kind. But it is not so easy to obtain the right sort, and keep up the standard. I think that it is most important our nurses should be girls of education and refinement."

"Of course they attend lectures?"

"The resident doctor usually lectures once a week to them on anatomy and physiology, and I on nursing. I also give a bandaging class once a week, so that they may know something about bandages when they go to a general hospital, and I am glad to say that this knowledge has been found useful."

"I suppose that sometimes you have bad cases?"

"Yes, and occasionally operations. The nurses at least get an insight into surgical work; and the sister in the nurses' corridor, who is herself an excellent surgical nurse, teaches antiseptic nursing to them whenever she has an opportunity. But, generally speaking, the work consists more of looking after the patients and carrying out rules than of ordinary nursing."

The Diet Question.

"Then they learn a good deal about diet and discipline?"

"Undoubtedly. A considerable amount of their time is occupied in carrying food to and from the wards. There is a wardmaid in each corridor to do the polishing, but the nurses see to all the food. We are very particular that the patients shall have their meals hot, and it is the duty of the probationers to take care that there is a cover on every plate. They also help the vegetables, and are expected to put the food on the plates in the most tempting manner."

"Are the patients forced to eat?"

"No; we do not force, but we do all we can to encourage them to eat. They are given very liberal portions of meat and pudding at dinner."

"But supposing they do not finish them?"

"I always, with the assistance of one of the sisters, carve at dinner, and see how much each patient eats. If I think the quantity insufficient, I report to the resident physician, and he exercises his discretion as to the treatment.

### Milk Compulsory.

"Although there is no compulsion about ordinary food, every patient is required to take a quart of milk daily."

"In instalments?"

"Yes, half a pint at breakfast, half at luncheon, half at dinner, and half at supper. Two months ago we paid £50 for the large steriliser in the corridor on the ground floor. It was put there as the kitchens are at the top of the building, and I wanted to see to it myself. Directly the milk comes it is put into the steriliser, and after being sterilised is measured out to the wards. Next in importance to open air and diet is the question of rest."

### Rest and Exercise.

"What are the arrangements?"

"The bell rings in the morning at 9 for rest, and the patients have to rest until 9.30. It rings again at 12, and rest is enforced until 12.30. From dinner the patients go to rest until 2, when the bell rings for recreation, which includes reading or writing, or playing games, such as billiards or chess. The men are allowed to smoke. At 3 exercise commences.

"That is an important feature?"

"Very. It lasts for an hour and a half. The exercise grounds are opposite the windows of the resident doctor, and the nurses have to see that the patients walk up and down the terrace during that time. It is frequently difficult to get them to do so. From 4.30 until 5 they go to their couches again, and then there is tea."

"Is there any rule as to the amount of butter?"

"The butter is always thickly spread on the bread and the patients can have just as much bread and butter as they like. Their friends are allowed to bring in jam or eggs for tea—nothing else. The nurses boil the eggs. After tea there is rest until 6 and then recreation until 7.45. After supper they have a bath or are rubbed, but all must be in bed at 9. The bell for getting up in the morning rings at 7.30, and breakfast, which is supervised by one of the sisters, is at 8.15."

### Weighing Parade.

"Another of the duties of the nurses," continued the Lady Superintendent, "is to administer cod-liver oil, which most of the patients take after their principal meals. Then there is weighing parade."

"How often does that occur?"

"Every Wednesday. In order to test their progress properly, each patient has to put on the clothes worn at the time of admission. One of the sisters superintends the business and registers the weight. As you can imagine, the patients are very anxious to know whether they have gained a bit. Sometimes they come to me looking so happy and say, 'Matron, I have gained three pounds.'"

### The Great Want.

"With all your beautiful grounds and admirable accommodation you do not possess a nurses' home!"

"No; I wish we did. The house at which eight of our nurses sleep is only a few steps from the hospital. The night-sister and nurses sleep here. We have space for a home and we only want money to build it. There is no doubt that it would be a great convenience."

### The Nurse in the Mission Field.

By a Correspondent.

Among the many who yearly qualify themselves to act as nurses, there are some who do not quite know what to do when their training is finished. They have not perhaps that inborn love for nursing, as an art, a science, or an occupation, which leaves a woman in no doubt of her vocation. Or perhaps, the necessity for earning a livelihood not being urgent, the question arises whether it is fair to compete professionally with those who have their bread to earn. Or again, perhaps, the course of training has made it clear that nursing is not really a sufficiently congenial task to be considered as an end in itself, or to be regarded complacently as a life's occupation. What then? The desire to be useful remains—the desire to do something in and for the world which in many cases prompted the choice of nursing as an outlet for philanthropic energy and an escape from a life of mere "passing the time."

### A Field in India.

To such as these I would suggest that a great field for usefulness lies open in India. We are all of us Imperialists nowadays, conscious of a new sense of kinship with our fellow-subjects of other lands, born of sympathy and fellowship in the public joys and sorrows of the last few years; we shall do well then to look beyond merely national and insular needs, when we are thinking how to be of use. We are all of us moreover longing to build a worthy memorial to the beloved Queen, whether in visible shape of stone or in the endowment for all time of a work which she held dear—provision for the nursing of the poor in their homes. We shall do well also to remember that her best memorial will be raised by lives of absolute devotion to duty like hers, and that as her care was not for the people of these isles alone, so this memorial of her should be seen among all her subjects. "Is not the greatest woman in the world dead? Has not her rule ended? Will not women therefore be worse off all over the world?" So people asked in India when the news came. Shall not we answer, "Nay; but because she lived all women are better off," and strive because she cared for Indian women, as for all her subjects, to do something to improve their lot?

### An Appeal to English Women.

We do not yet sufficiently realise what an enormous amount of preventable suffering is silently born by the women of India, who are of like flesh and blood with ourselves. It is preventable suffering, because it is suffering caused in a great degree by ignorance and prejudice. But it is suffering which neither Royal decree, nor Acts of Parliament can hinder. Love, patience, persuasion, sympathy skill, education will in time win their way against ignorance, custom, and prejudice. To remedy present suffering and at the same time to help towards removing the causes of suffering is surely a splendid task. And this task seems specially committed to Englishwomen at the present time. To women, because only by women can the secluded women of India be approached; to Englishwomen because the Government of India is entrusted to England, and England is responsible for the well-being of her subjects, and at the present time, because now, as never before, a spirit of inquiry is stirring amongst the peoples of India, old customs are weakening, and the time is ripe for the acceptance of more liberal ideas, especially with regard to the position and education of women. Are there not some amongst us to whom this task appeals?

Examples of Suffering.

Here are one or two examples to show the kind of preventable suffering I have in mind, gleaned from the report of the S.P.G. and Cambridge Mission to Delhi. "One case was that of a little Hindu child wife under 12, with extensive bone disease of the leg. . . . Early marriages and their unhealthy surroundings are largely responsible for these cases of bone disease and wrecked constitutions in young girls; it seems as if the one idea of the parents is to get free from the burden of keeping their daughters as early as possible, and the 12 year old law is a dead letter here in Delhi." Again, "It is disappointing in many cases to find the patient, to whom one may have been called in the middle of the night, moribund already, though timely help would have saved her—or one has to waste precious hours in vainly trying to persuade the husband and friends to allow some urgent operation to be done; all expostulatious being met with the hateful but unanswerable phrase, 'Io hoga, so hoga' ('what will be will be'). The blank wall of Kismet meets one here as elsewhere in the east, paralysing all effort and progress, and condoning the most appalling cruelty. Truly it needs some patience to stand by and see women and girls who could be saved from suffering and death, slowly tortured, and losing limbs or life, owing to 'religious' prejudice, and the ignorance of men which no mere instruction in schools or colleges seems able to dispel.' Once more: "In one large Hindoo house a young wife of sixteen had long been suffering from internal abscesses. Going one broiling July day to visit her as usual, it was to find that she had turned faint in the early morning (owing to being neglected all night by the lazy women in the zenana), and so, thinking she was dying, the men had thrown her from the bed, and bumped or dragged her down the steep stone staircase to the lower floor, where she lay close by the evil-smelling refuse bin, ready for removal when dead. In this, however, she disappointed them, and they had presently the trouble of carrying her all the way back again to the zenana; but at the cost of what terrible suffering to the girls and women are such scenes as this enacted! They make one realise the essential difference between east and west, the Christian and non-Christian civilisation; in the newest Christian homes one never would find a daughter so treated, even among the poorest or most ignorant of the converts."

A Queen's Jubilee Nurse at Cawnpore.

These are incidents noted in the course of their daily work last year by English ladies who are spending their lives in ministering to Indian women. They are not by any means exceptional cases. The Dufferin Hospitals and Medical Missions in their several ways are doing a great deal to alleviate this suffering. In Cawnpore the Women's Mission Association, the women's branch of the Society for the Propagation of the Gospel, has quite recently built and equipped a beautiful hospital at which in 1900, the first year of its existence, the attendance of out-patients exceeded 10,000. In that hospital, a Queen's Jubilee nurse is in charge of the wards and is training a staff of native Christian nurses. She gave up her career at home and offered herself as a missionary because to her the call of India seemed irresistible. She writes that she is sure that if only readers of the Hospital Nursing Mirror knew of the W.M.A. Hospital at Cawnpore, its needs, and its great opportunities for good, they would want to help it, by endowing or supporting a nurse, a bed, a cot, or by making garments, or bed-clothes, sending pictures, etc.

An Opportunity for Service.

But my object in writing is not chiefly to plead the cause of medical missions, nor to appeal for nurses for India as such. It is rather, as I said at the outset, to suggest a splendid opportunity of service to those who do not care to follow the profession of nursing solely and entirely. To live as a missionary among these people in India, to devote life and energy to their service, to show them sympathy, to bring light into their dark places, is, I repeat, a splendid task. It calls for gifts of mind and temper, and for a consecration of will which it is beyond the scope of this paper to describe. What I would urge here is that nursing experience in addition to other qualifications, will never be thrown away. It will often be a passport to the confidence of secluded women in zenanas, and of ignorant country folk, and always a great help in the care of children. In many districts in India it is still no uncommon thing to be 50 miles from efficient medical help: therefore it stands to reason that in such places a woman who has nursing experience, given equal qualifications in other respects, is likely to be twice as useful as one who has not. For this reason it seems well to suggest that those who have been trained as nurses should ask themselves if it is out of the question that they should also be missionaries? If any readers of this article would like further information on any of the subjects I have touched upon, they have only to write to the secretary of the Women's Mission Association, S.P.G., 19 Delahay Street, Westminster, who will gladly give all particulars.

### Sick Nurses for Shipboard.

By a Voyaging Nurse.

(*Concluded from page* 305.)

Nowadays the food on a good line of steamers is usually beyond reproach, and an invalid will have little just cause for complaint as to the range of dietary. Often the ship's cook is a capital hand at broths, beef-teas, and custards. But there is so little provision made in a ship's routine for the serving of meals in the cabins that an invalid necessarily suffers long and weary waits between meals.

The Régime for Invalids.

Naturally enough, the ship's regulations are made on the assumption that the passengers are well and strong. The sick department so far has scarcely been taken into account. While the saloon meals are being served the cabin stewards and stewardesses are commonly taking their own meals. This is just the time when they legitimately count upon a little leisure for themselves. It is delightful even to think about and theorise on the little comforts and changes in the *régime* for invalids that a practical ship's nurse would inevitably make. Her position need not necessarily involve very hard work. No rough duty would devolve on her. There are plenty of helpers on board a passenger steamer—especially on those lines which employ native stewards—but it would be her province to organise and superintend the comforts and care so essential to persons suffering or convalescing from the wasting and exhausting diseases peculiar to tropical and bad climates.

Cases for Nurses.

On my last voyage two confinements took place within a day or two of one another. Both were steerage passengers. There was nobody on board save the doctor and myself who had any professional knowledge of the care of such cases. And these two women, in addition to the misery of being confined in close hot cabins in the Red Sea, had no nursing care beyond the ignorant and casual ministrations of fellow-passengers and the very limited superintendence that a steerage stewardess could spare out of her busy life and many duties. A lady among the saloon passengers had a miscarriage in mid-ocean—these sort of things being specially liable to occur in the tropics, especially amongst women who have lived long in the East or are suffering from dysenteric complications. The lady in question had no maid with her, and tried vainly at Colombo to obtain a trained nurse or even a native ayah. I could give endless examples of this nature to illustrate the need of a qualified nurse on every big passenger steamer. Even between Liverpool and New York I have seen much avoidable suffering arise from the absence of any skilled attention in cases of illness. It is true that the need of a nurse on board the quick crack Atlantic liners is much less than on the steamers coming home from our unhealthy Colonies and the Far East and involving voyages of many weeks' duration. But a good deal may happen in the way of grave illness in the space of a week, many dangerous diseases may set in and run their course between Queenstown and New York Harbour.

### The Question of Children.

That steamship line plying between unhealthy Eastern ports and home which first has the enterprise to appoint a trained nurse on each of its boats will certainly ensure a large measure of popularity. There will be a "run" on its cabins on the part of invalided and delicate folk bound for home and health. And it would assuredly prove of the utmost comfort to parents bringing sickly children from tropical stations to be able to count on the care of a certificated nurse should their little ones be seized by those terribly sudden illnesses so apt to attack tropical children *en route*. Indian children coming home are liable to serious chills owing to their transition by quick stages from a very hot to a cold climate. Many such children are unavoidably sent home with only an ayah to look after them on the voyage. Now the ayah of India and Ceylon—like her sister amah of China—proves a most devoted nurse, but she does not know much about the care of sick European children. So far as affectional devotion goes, she will do everything for those under her charge. But should one of them develop pneumonia, enteritis, or dysentery on board the average ayah does not possess the technical knowledge necessary to such cases. With a trained nurse to direct and show her what to do the ayah will carry out instructions admirably. In the absence of such supervision she will probably prove most helpless.

### A Ship Nurse's Duty.

Of course it is not to be expected that one ship's nurse could do the routine nursing of a steamer load of passengers. But she could point the nursing way. She could see that subordinate stewards and stewardesses carried out her instructions; the higher and more subtle nursing duties she would perform herself. There is no doubt in my mind that this reform will shortly be accomplished. It is only a matter of a little waiting for public opinion to formulate itself and then passengers will no more ship on a nurseless than they would on a doctorless ship. It would form part of a ship nurse's duty to accompany the doctor on his daily rounds and help him with the numberless dressings, vaccinations, etc., which have to be carried out in the steerage department. With a full steerage the doctor is often worked to death; he has nobody to help him in the hundred little detail duties which are all in his day's work.

### Impracticable Suggestions.

In talking the matter over with several steamship company authorities, I find most of these agree that the appointment of a ship's nurse would be an excellent step. Some of them have suggested that nurses going to and from tropical stations might be granted free passage on consideration that they gave their nursing services to the passengers on board. To the nursing mind many objections to this plan present themselves. A nurse going out fresh to the tropics would be in good condition and able to look after the few cases of illness arising on an outward voyage. But such a nurse returning on leave after some years in a trying climate would certainly not be physically strong enough to undertake the heavy freight of invalids carried on the homeward voyage. So far as the East is concerned a nurse's duties would be very light going outwards and the post an easy one. But usually there would be little leisure on the return journey. Ship's nursing could not be carried out satisfactorily in this scrappy fashion with a new nurse shipped for each voyage. Some sort of permanence would be necessary to success. To be a good sailor would form an essential qualification, and to be of real value to her patients a "sailor nurse" must understand the care of the special diseases likely to be prevalent among passengers from hot climates.

### A Miserable Experience.

Perhaps only those who have themselves sea-voyaged in a sickly state of health, in a tropical country, can appreciate at its true value all the wretchedness and hardship that may be comprised in a prescription to "try a sea cruise." I can afford to laugh now at the memory—though it was a very miserable experience at the time—of forming one of a company of several invalids sent from Colombo on a slow steamer calling at all the ports on the Coromandel coast up through the Bay of Bengal and on to Calcutta. We were a sorry, helpless conglomeration; no doctor is carried on a coasting boat, there was not even a native woman on board, only "black boys." It is considered a sovereign remedy in the tropics to try a cruise of this kind, and if no benefit be obtained the next stage in the prescription is to be ordered home. We all voted the coasting remedy worse than the disease. On coasting steamers there is no refrigerator, and fresh food supplies are rare; ice is almost unknown. From the invalid point of view there is no sort of comfort possible. Several among us left the ship at the first healthy port of call, which was about twelve days off Colombo, and found at a delightful sanatorium resort those comforts and conveniences of dry land which are impossible of attainment by the sick at sea. My personal experience, added to observations of invalids on many ships and many voyages, has convinced me that sick persons had better avoid the sea unless they can afford to take a nurse with them.

### Native Nurses Impossible.

Ordered home from the tropics to save their lives they have to face with what fortitude they can the drawbacks and miseries of a nurseless state during the voyage. No doubt the time will come when there will be, at any rate in the larger cities of the tropics, a considerable number of hospital-trained native women whose nursing services will be available on homeward voyages. But that is not yet, and the rate of training is very slow. Meanwhile, the carrying of a qualified European nurse on each of the big liners of the East would be an admirable and practical method of mitigating the sufferings of the sick at sea—of sick men especially, who necessarily travel home alone. These would assuredly rise up and call blessed any steamer which carried a qualified sailor nurse.

### To Nurses.

We invite contributions from any of our readers, and shall be glad to pay for "Notes on News from the Nursing World," or for articles describing nursing experiences, or dealing with any nursing question from an original point of view. The minimum payment for contributions is 5s., but we welcome interesting contributions of a column, or a page, in length. It may be added that notices of entertainments, presentations, and deaths are not paid for, but, of course, we are always glad to receive them. All rejected manuscripts are returned in due course, and all payments for manuscripts used are made as early as possible after the beginning of each quarter.

### Echoes from the Outside World.

### AN OPEN LETTER TO A HOSPITAL NURSE.

The horror with which the news of the attempted assassination of President McKinley was received, on Saturday, was universal, and the sentiments of sympathy expressed by King Edward, by "William, R.I., and Victoria, R.I." (the Emperor and Empress of Germany), and by other Royal personages, was echoed by every man and woman throughout the civilised world, with the exception of a handful of miserable Anarchists who doubtless rejoice that a blow has once more been struck at the head of a nation. The point which strikes me most forcibly with regard to Czolgosz's crime—Niemann appears to have only been an *alias*—is that he states his first incentive to crime came through his having attended the lectures of a woman. For five years he had associated with Anarchists, and had become morose and envious, but he had never contemplated murder until he went to hear Miss Emma Goldman, in Cleveland. According to his own statement, "She set me afire with the doctrine that all rulers should be exterminated. . . . Her words went right through me, and I made up my mind that I would have to do something heroic for the cause I loved." Twice a day on Tuesday, and again on Wednesday, the wretched man went to the Exposition, Miss Goldman's speech still "burning him up," till at last on the Friday the chance came. It is not surprising that the arrest of Miss Goldman followed the event. She is said to be a good-looking Russian, and at the beginning of the Boer War she lectured in Clerkenwell Road against royalty, religion and society to a packed hall and no one attempted to stop her. When the arrest was made she disclaimed all knowledge of Czolgosz and his crime; but subsequently stated that he had a few words with her recently and told her that he had heard her lecture and wanted to know her. "That was all."

Up to the present the President has made satisfactory progress. His calmness and bravery does much to encourage his physicians, and he in turn is cheered by the love and devotion of his wife. Those who have had the privilege of seeing Mrs. McKinley say that it is almost impossible to believe that it is such a short time since she herself was a serious invalid. Now that need for action has arisen, she has braced herself to meet the trouble and has borne up with really remarkable fortitude. Though she spends much time by her husband's bedside she cannot afford to neglect her own health, and she constantly takes tonics and sedatives. Several times she has been out driving with her cousin in the afternoon, returning rested and refreshed. When the disaster first happened some surprise was expressed that on such a public occasion as a reception there were no special precautions taken for the safety of the President. But it now appears that every precaution had been observed. Two private detectives were quite close, one almost touching him, the other just opposite. The reason why the second was not in his usual place, a little to the left of Mr. McKinley, so as at once to note all who approached, was because Mr. Milburn wished to introduce people as they came up, and thus occupied that position himself. Upon such apparently trivial matters do great events hinge!

You will remember that Mr. McKinley is by no means the first President of the United States to suffer from the hand of the assassin, though it is to be hoped upon this occasion that the result of the dastardly act will be less fatal. In 1865, when at last the end of the sad Civil War was near, and America was rejoicing, President Lincoln went to the theatre one evening to see "Our American Cousin." Suddenly, in the middle of the play, a shot was heard and a man leaped out of the President's box brandishing a dripping knife. He had stabbed one of the other occupants of the box, after placing the pistol close to the President's head and shooting him so that the bullet entered the brain. The wounded President was carried to a house across the street and died early next morning. The murderer was an actor named John Wilkes Booth, who was desirous to "avenge the South," he said. Sixteen years later, at Washington Railway Station, a disappointed office-seeker, a Frenchman named Guiteau, fired at President Garfield. The first shot only grazed the victim's sleeve, the second unfortunately entered his body. For two months Mr. Garfield hung between life and death. A day of national supplication for his recovery was appointed, and at one time it seemed likely that his life would be spared. But blood-poisoning set in, and after many weeks of suffering, bravely and cheerfully borne, President Garfield passed away. Those whose memories can go back sufficiently far will recall that Queen Victoria—who had had daily bulletins sent to her—ordered the Court to go into mourning for a week upon his death, and forwarded a most beautiful and touching message to Mrs. Garfield.

We have of late been so far removed from the land of theatres that I was seized with a sudden desire for excitement and to see what our ancestors used to call "play-acting." A casual glance down the amusement column of the newspapers decided me at once where to go. I have always been a great admirer of Thackeray in common, I suppose, with half the civilised world, and I was particularly anxious to make the acquaintance of the renowned "Becky Sharp" behind the footlights; so we went to the Prince of Wales's, never doubting that as it was a new piece and in the dull season we should have no difficulty in securing a choice of seats. But if we had been a little later we should have had to go elsewhere, for the house was quite full—stalls and pit, dress-circle and gallery—and I wondered where all the people came from. It shows conclusively how soon a rumour of success gets wind, for there is no doubt that "Becky" is going to hold her own for a long time to come, partly because of the national love for "Vanity Fair," and partly because the play is most interesting and extremely well acted all round. I had no idea that Miss Marie Tempest could summon to her aid such real dramatic power. The fun, the demureness, and the pertness of the first part of the performance were, I knew, well within her power, but the force and the reality of the last sad scene were quite a revelation. Mr. Gilbert Hare as the Marquis of Steyne is as good as his father would have been in the same part; Mr. Leonard Boyne, successful enough as the good-natured if rather foolish Rawdon Crawley, and if he laughed less would be more impressive; and Mr. Holman Clark is quite as sanctimonious in the first act as the representative of Pitt Crawley ought to be. The scenery is excellent, and nurses who can get a few hours off duty will thoroughly enjoy the time spent with old friends now—for the first time, I believe—placed upon the stage.

Nurses who love the invigoration of a "blow on the briny" may like to know that the New Palace Steamers will make a special trip to Dunkirk on the 17th or 18th instant (whichever date is fixed for the Naval Review) to view the French Fleet which is to be reviewed by the Tsar of Russia. The *Marguerite* will leave Tilbury about 8.30 A.M., and first-class special trains will start from Fenchurch Street at 7.30 A.M. and from St. Pancras at 7.5 A.M. The fare for the return journey is to be a guinea, but only a limited number of tickets will be issued. The trips to Boulogne and Ostend will continue up to the 19th, and those to Margate until the 23rd, but the boats to Ramsgate have already ceased to run.

### The Irish Nursing Question.

BY A DUBLIN CORRESPONDENT.

A PECULIAR feature of the conference of delegates from Poor Law Unions in Ireland, just held in Dublin to consider the question of nursing in Irish Workhouse Infirmaries—a full report of which appeared last week—was that no one present seemed completely to understand the situation. Mr. Mooney, the chairman, a most experienced and competent guardian of the poor, and chairman of perhaps the leading Poor Law Board of the country, declared that "he had no particular knowledge about the matter himself," and that he had not had time to go into it very closely. There was the same want of grasp, and of accurate information, all through the proceedings. The report of the speeches at the conference, therefore, gives very little idea as to how matters stand, and I propose, so far as possible, to supply the omission.

### THE ORDER OF FEBRUARY 4TH.

On February 4th last the Local Government Board issued an order to the unions which a large number of them objected to as interfering unnecessarily with their authority. The petitions of the objecting unions were considered by the Privy Council on May 14th and 15th, and most of the evidence put before it dealt with the question of nursing. Counsel for the Armagh Guardians, whose case was taken first, stated that formerly the nurse was supposed to be an assistant to the medical officer. Later, however, by a new regulation the nurse was given the dignity and status of an officer. No objection was taken by the Armagh Board of Guardians, "on the contrary, they at once acceded to the request of the Local Government Board, and they appointed a day nurse and a night nurse, with such qualifications as the Local Government Board prescribed. What they substantially complained of in this case was that every assistant in every poorhouse in Ireland should have such qualifications as the Local Government Board required." At the instigation of the Local Government Board, counsel went on to say, a system was adopted throughout Ireland under which there should be one trained nurse for the day and one trained nurse for the night. The Armagh Board of Guardians had appointed two qualified nurses with the consent and approval of the Local Government Board. They then started upon a plan of getting strong, healthy girls from the locality to come into the workhouse infirmary as assistant or probationary nurses. These girls were trained under a very skilled medical officer, and they had also the advantage of being trained under a skilled nurse. The Local Government Board recognised this training in the workhouse as being a qualification for the position of head nurse, and when the office fell vacant in Armagh one of these probationer nurses was so recommended by the medical officer that she was appointed. It became necessary to supply her place with another person from the locality, but the Local Government Board insisted on sending a nurse down from a Dublin institution. The Guardians, however, would have nothing to do with her; and then the Local Government Board issued the Order applying to the whole of Ireland. To this statement counsel for the Local Government Board replied that case after case had been reported of grossly inadequate nursing arrangements, and grossly inadequate hospital attendance, that the board had found it necessary, in the interests of the sick poor, to ask the Council to restore to them the full jurisdiction they possessed under the statute. Dr. Bigger and several other Local Government Board Inspectors gave evidence in support of this contention. Dr. Bigger stated that there had been great difficulty in getting the guardians to appoint efficient nurses. There were 28 workhouse infirmaries in which there was no trained nurse whatever. There were 40 or 45 in which the staff was manifestly insufficient. Mr. Agnew, another inspector, stated that in 1893 he called attention to the fact that there was no night nurse in the Armagh Workhouse. In July 1894, an inquest was held on the body of an inmate. In that year he had to report that there was only one paid nurse for 60 patients and 23 female lunatics. He discovered that the meat supplies for the week were sent in at the same time and put in pickle. No nurse, he added, would come to the Armagh Fever Hospital; it was in such an insanitary condition. Mr. Micks, formerly an inspector, now a member of the Board itself, mentioned that when a vacancy occurred in Derry Union for the office of nurse, he urged the Guardians to appoint a trained nurse; but they thought the idea a "fad," and appointed a farmer's daughter, who had never seen the inside of a workhouse. The number of sick in the infirmary at the time was from 55 to 65. The attitude of the Guardians with respect to this matter, he declared, was entirely due to want of perception of the necessities of the case, and to a desire to save the rates. On this point Dr. Smith, of the Naas Union, said that Guardians could not be blamed for not appreciating nursing in the modern sense, as it was only recently that medical men themselves had begun to appreciate it. The result of the evidence was that the Privy Council disallowed the Order, which was largely concerned with general administration, as it stood. "But they report," says the *communiqué* to the press, "that evidence has been given establishing to their satisfaction the necessity for some rule or order which will make better provision for efficient nursing."

### THE ORDER OF JULY 5TH.

As a consequence of this decision the Local Government Board issued, on July 5th, the Order which has occasioned the present agitation. In the first article of this Order the prohibition of pauper nursing made in the Order of 1897 is reissued, with some verbal alterations, and under the article it is required that no pauper shall be employed as an attendant in the sick, lying-in, or infant wards of the workhouse without the approval of the medical officer, and unless such pauper act under the supervision of a paid officer employed in the ward. No serious objection seems to have been taken to this provision in any of the unions. As several of the delegates at the conference said, "nobody wants to go back to the old system of pauper nursing." All the misunderstanding to which I referred at the beginning seems to have taken place in connection with the second article of the order which defines what are "trained" and what are "qualified" nurses. I have reason to know that the Local Government Board people were amazed at some of the statements made in this connection at the conference. For instance, as your report of last week shows, one important speaker declared that, with sufficient money, a nurse could get certified in some of the recognised hospitals in twelve months. Another speaker, who should have known what he was talking about, mentioned the possibility, under certain circumstances, of getting a certificate in three months. Rule 2 of the Local Government Board Order of July 5th disposes at once of these statements, so far as the Local Government Board is concerned. It reads as follows:—

"ARTICLE 2.—(*a*) In this order the term 'trained nurse' shall mean any person who has resided for not less than two years in a general clinical or other hospital recognised by us, and who, after examination, has obtained from such hospital a certificate of proficiency in nursing."

### THE ALLEGED MONOPOLY.

Several delegates referred again and again to the "monopoly of a few Dublin hospitals." As a matter of fact the Belfast Workhouse Hospital has been training for the last couple of years, and its nurses are employed as trained nurses all over Ireland. Cork, Limerick, and Galway might, too, be in the same position if they thought it worth their while to work up their teaching efficiency so as to be recognised. There is another point about this article of the Order which was overlooked by the conference—the provision, namely, with reference to what are called "Qualified Nurses. Here it is—

"(*b*) The term 'Qualified Nurse' shall mean any person who, after examination, has obtained a certificate of proficiency in nursing from any

(1) Public general hospital; or
(2) Workhouse infirmary and fever hospital or
(3) Nursing institution; that may be recognised by us as an efficient school for medical and surgical nurses."

This, it will be seen, still further reduces the "monopoly," and I understand that quite a number of county and workhouse hospitals might easily be themselves recognised in this connection. But to still further show that there is no Dublin monopoly, it is only necessary to mention that nurses with certificates from any properly recognised hospital in Great Britain, the Colonies, or the United States are in the same position under the rules of the Local Government Board as a nurse fresh from the banks of the Liffey. However, the conference has decided that there is a monopoly, and the permanent committee it has appointed will likely approach the Chief Secretary, or the Local Government Board itself on the subject. It is said that Mr. Wyndham is inclined to view a widening of the training powers of the smaller rural hospitals with favour, and perhaps he may in some way modify the Order. But the Local Government Board itself is quite certain that the Order goes as far in that direction as is good for the interest of the sick poor. Meantime, let me add, in conclusion, that under the new regulations the nurse and the nursing staff are no longer under the control or supervision of the matron, or of any other officer except the medical officer and (for general disciplinary control only) the master of the workhouse. "This change," says a circular of the Local Government Board, "is made because, on the introduction of trained or qualified nurses, it has become most undesirable that even a nominal control over the sick wards should be in the hands of persons without training or qualifications in the management of an infirmary or hospital. The management of the sick wards under the guardians will accordingly be in the hands of the medical officer, to whom the guardians should look for the satisfactory condition of their infirmary and hospital. . . ." In the absence of the medical officer, the nurse of the workhouse would exercise general supervision and control over the sick, and over the nursing and menial staff.

### Everybody's Opinion.

[Correspondence on all subjects is invited, but we cannot in any way be responsible for the opinions expressed by our correspondents. No communication can be entertained if the name and address of the correspondent is not given, as a guarantee of good faith but not necessarily for publication, or unless one side of the paper only is written on.]

### MEALS UPON THE STAIRS.

"E. L. M." writes: The story of the two nurses at the school having to take their meals on the stairs reminds me of a case of tracheotomy I had some years ago, at a little second-hand furniture shop in the country. I was not able to leave the sick room for two days, and great was my surprise on doing so to find my dear little grey bonnet reposing on a very disreputable pair of boots on the more than dusty uncarpeted stairs. I suppose I must have looked something, but I did not speak; for the mother came forward and said, "Oh nurse, I thought that was a cap you put on to clean the grate in, so I put it ready for you." The same lady came to me on my departure, and said while holding my hand and with tears in her eyes, "Thank you nurse for all you have done for us, my one great consolation is, had my little girl lived she might have taken to drink or had to be a nurse, and although you may not have means you might any day be mistaken for a lady, so do come and stop with us whenever you are needing a holiday."

### WORKHOUSE NURSING.

"Miss C. J. Wood" writes from the Nurses' Hostel, Francis Street, W.C.: The fact that a deputation from the Workhouse Nursing Infirmary has been received by the President of the Local Government Board, and that its statements and suggestions have been listened to, is a cause of congratulation to all who have the good of the pauper sick at heart. But, unfortunately, one is accustomed to the official civility which seems so deeply interested whilst all the time the case is prejudged and decided before the council comes into court. The country must not be satisfied with the policy of drift. The scandal which clings around the nursing of the sick pauper has been admitted on all sides, and it is time that something effective was done. Now that an intelligent nursing order has been issued in Ireland we may take hope for the "adjacent isles," and if only Mr. Long would make some similar decree from Whitehall many of the difficulties would be cleared away. I hope that the Workhouse Nursing Association will peg away until the wrongs of these silent suffering sick paupers have been righted.

"A Certificated Nurse" writes: Dr. Humphreys, in his letter, quotes the old saw that "outsiders see most of the game," but I doubt very much whether outsiders are at present seeing much of the game that is being carried on at the Croydon Union Infirmary. Five nurses have sent in their resignations during the week, which shows the system of training is somewhere radically wrong. Else, why these resignations, making eleven in all, within the past two months? Evidently, more are imminent. Up to the present moment no inquiries have been made as to the reason, and slowly but surely, the nurses who have had any training (under Miss Julian) are resigning, and the lives of nearly 400 patients will be in the hands of a few inexperienced probationers. Dr. Humphreys' scheme is in many ways admirable. Nurses would receive thorough training, and would not, when half-way through their probation, have to give up, because of the inefficient training; and being under one central body, would not be subject to the petty injustices of an individual body of guardians.

### THE WEEDING OUT PROCESS.

"W. W." writes: The subject of "weeding out" is of great interest not only to the profession, but also to the patients. May I suggest, as a superintendent and a sufferer, that the difficulty might at least be lessened by the superintendents of private nursing homes joining together, either personally or by post, exchanging lists every six months, containing the names of the unsatisfactory nurses. Then the black sheep could not go from "home to home" without the knowledge coming to the superintendent. My experience has been long and varied, but I find that the nurse to be dreaded most, is one who considers herself too grand to do the real nursing, and only wants to nurse the rich. What can be more contemptible in a woman and a nurse than boasting of her family position, number of servants kept, etc., telling the unfortunate patient that she is more fit for the infirmary than for a private nurse? The foolish talk of the too grand nurse is humiliating for anyone to listen to, whether in health or in sickness. Although we can say with all truthfulness that the profession holds some of the best and noblest of devoted women, we have to acknowledge the fact that the unworthy few do much to lower its tone. Still, at present, we do nothing. Let the "weeding out" question appeal to all as if it were a personal matter, then, indeed, we may hope to see the problem solved.

### HINTS ON THE MANAGEMENT OF CONVALESCENT HOMES.

"The Matron of a Yorkshire Convalescent Home" writes: In answer to "O. C. E." *re* my paper "Hints on the Management of Convalescent Homes," I would remind her that I did not say I took over the bread-baking, but the making of it, which means the kneading of the dough—the baking is done after this has had time to rise, and by mid-day my baker, having helped with the morning scrubbing and cleaning, saw to the baking of the loaves. In one and a half hours it is possible to knead a large supply of dough, and I fancy any matron and assistant willing to rearrange the routine work, though necessarily interfering with the leisure time usually taken, could in nine cases out of ten do as we did, and take the extra work without neglecting regular duties. Then as to one "lecture being sufficient to prevent waste ever since," I think my questioner could not have grasped my statement that "each admission day I tell the patients collectively that the committee invite them to eat heartily at every meal, but at the same time mention that they are as emphatically asked not to waste or allow others to waste any food whatever." There is a saying "A little leaven leaveneth the whole."

### PRIVATE NURSES' FEES.

"POLICY 3633" writes: So many letters have been written on the disadvantages of Nursing Institutes or Homes, that it occurs to me to state some of the advantages which have influenced me in preferring to work for an institute during a great part of my nursing career. First may be mentioned the business part of the arrangements. Frequently, when being interviewed by ladies for maternity work, they have remarked "Your terms are very high." It was a great satisfaction to be able to say, "I have nothing whatever to do with that, the fee is an arrangement between yourself and the institute, I undertake the work and the institute pays me my salary." On the other hand, when working on my own account, I have sometimes unwisely consented to reduce the really reasonable fee, and on one occasion after doing so, I was kept waiting nine weeks beyond the date of my engagement, by which I lost a case at full fees, and only received the reduced fee and not one penny for all those weary weeks of waiting; preferring loss to law courts I had no redress. How frequently nurses write to THE NURSING MIRROR asking advice about such cases, or how to recover fees or parts of fees, is proof that such things are only too common. There are people who will treat a nurse working on her own account in a way they would never dream of treating an institute, and by working for one we are relieved of these worries. The printed rules sent with each nurse are useful in houses where the nurse is expected to do without sleep, food, or fresh air. Also on returning from a case it is nice to find our bed and meals ready without trouble or forethought on our own part. Companionship again is no small advantage. I have many pleasant recollections of congenial companionship and cheerful intercourse with other nurses during the few days' waiting time between cases. Some sad experiences have come under my notice of really good nurses who, anxious to receive their own fees, have started on their own account, and who have been reduced to the verge of starvation, and after parting with watches and trinkets, were very thankful to return to institute life. The suggestion of "A Sympathiser" for nurses to combine, and form "Nurses' Club Homes," is good if it can be carried out on systematic and business lines, and may prove of advantage to nurses, though there are many possibilities to arrange for.

### TRAINING IN A MATERNITY HOSPITAL.

"L. C." writes: In contrast with the article by your correspondent, I venture to send you an account of the very happy six weeks I spent during my training (I believe in the same hospital) and which came to an end all too soon. Much too excited to think of the dull surroundings, I rang the door bell which was answered by the porteress, who left me in the care of a nurse. Common sense told me I should not be allowed in the wards in private dress, so I went in uniform and a few minutes sufficed to make me ready to follow the nurse who had kindly waited to take me down to tea. In the old part of the building a large room curtained off was used as a nurses' bedroom, but in the new wing to which I was taken there were divided cubicles, with bath (h. & c.) on each floor. There was plenty of good food in great variety, well cooked, and milk or ale for dinner and supper at a small extra payment, the matron carving and dining at the same table. After evening prayers we could retire to bed or stay in the dining-room with use of piano. A thoughtful nurse told me I might be called up in the night to a case and I must be dressed in three minutes to be there in time—which is easily managed if your heart is in your work, for I was never later than second to appear in the labour room, sometimes twice in one night; only the lazy ones arrive to find the baby born. Next morning at 8 o'clock my turn came; at the sound of the bell we all left our work and went quickly but quietly to the labour room. After assisting (or hindering?) the matron during the delivery, I cleared away every utensil used and found sister waiting to show me how to wash the baby. Everything is done to give you confidence and help in performing the act of bathing a new-born babe. The stores are served out twice daily, being the necessary things for the comfort of the mother and baby. After this each nurse cleans and disinfects every utensil used for her patient, including the polishing of the bed pan, which if you take a pride in making yours the brightest in the ward, your "poor hands" are soon forgotten. The night work is done by two nurses and one super; the meals for the patients, the super, and ourselves are prepared in a handy little ward kitchen on the same floor. We had little time to make use of it as a sitting-room, there being plenty to do, including answering the door-bell to admit patients, who want as a rule immediate attention. The patients have a special room called the receiving room, to remove their outdoor garments and change into more suitable attire. I cannot understand any nurse feeling sleepy on night duty; there is so much to be done, and if she realises the responsible part she ought to take, and puts her whole heart into the work, morning comes before it is possible to realise it. The matron and staff do all in their power for the comfort of both patients and nurses, and I trust that the latter will do their best on leaving to be a credit to their training school.

### Appointments.

CITY ISOLATION HOSPITAL, NOTTINGHAM.—Miss Florence Weddall has been appointed night superintendent and deputy matron. She was trained at St. George's Infirmary, Fulham Road, London. Subsequently she did temporary duty at Cork Street Hospital, Dublin, and the Nurses' Cooperation, London, and was charge nurse at the Bristol Royal Hospital for Children and Women.

ECCLESALL BIERLOW UNION INFIRMARY.—Miss Rose Wills Pascoe has been appointed superintendent nurse. She was trained at King's College Hospital, London, and has since been superintendent nurse at Middlesbrough Union Infirmary.

HAMPSTEAD WORKHOUSE INFIRMARY. — Miss Ethel Florence Joy has been appointed charge nurse. She was trained at Worthing Hospital, and has since been staff nurse at Greenwich Infirmary, and charge nurse at Islington Infirmary.

ST. MARY'S, ISLINGTON INFIRMARY.—Miss Lilah East, Miss May Yule, Miss Eliza Wilkinson, Miss Bessie Teesdale, Miss Mary Read, Miss Minnie Dix, and Miss Lucy Everett, have been appointed sisters; and Miss Sarah Garrett, Miss Florence Ellworthy, and Miss Ellen Tibbles have been appointed staff nurses. Miss East was trained at Tottenham Hospital, and has since been sister at the South City Infirmary, Dublin; Miss Yule was trained at Mill Road Infirmary, Liverpool, and has since been charge nurse at the South-Western Free Hospital, London; Miss Wilkinson was trained at Chorlton Infirmary, and has since been staff nurse at Islington Infirmary; Miss Bessie Teesdale was trained at St. Saviour's Infirmary, Dulwich, and has since been charge nurse, and engaged in private work at Mentone; Miss Read was trained at St. Thomas's Hospital, the East London Hospital, Shadwell, and Monsall Fever Hospital, and has since been sister at Bethnal Green Infirmary; Miss Dix was trained at Edinburgh Royal Infirmary, and has since been ward sister at Hull Royal Infirmary; Miss Everett was trained at Hull Royal Infirmary, where she was afterwards theatre sister; Miss Garrett was trained at Newport Infirmary; Miss Ellworthy at St. Bartholomew's Hospital, Rochester; and Miss Tibbles at Greenwich Infirmary.

### For Reading to the Sick.

"THE HEAVENS DECLARE THE GLORY OF GOD."

THE works of God above, below,
Within us and around,
Are pages in that Book, to show
How God Himself is found.

The glorious sky embracing all
Is like the Maker's love,
Wherewith encompassed, great and small
In peace and order move.

Thou, Who hast given me eyes to see
And love this sight so fair,
Give me a heart to find out Thee,
And read Thee everywhere.—*J. Keble.*

More and more stars! behold yon hazy arch
Spanning the vault on high,
By planets traversed in majestic march,
Seeming to earth's dull eye
A breath of gleaming air: but take thou wing
Of Faith and upward spring:—
Into a thousand stars the misty light
Will part; each star a world with its own day and night.

Not otherwise of yonder Saintly host
Upon the glorious shore
Deem thou. He marks them all, not one is lost;
By name He counts them o'er.
Full many a soul, to man's dim praise unknown,
May on its glory throne
As brightly shine, and prove as strong in prayer
As theirs, whose separate beams shoot keenest thro' this air.
*J. Keble.*

The invisible things of Him from the creation of the world are seen, being understood by the things that are made, even His eternal power and Godhead.—*Rom.* 1, xx.

"Man could never have framed a name for God, unless Nature had taken him by her hand and made him see something beyond what he saw, in the fire, in the wind, in the sun, and in the sky."—*Max Muller.*

"God has created the heavens and the earth and the sea, and has most wondrously and beautifully adorned all nature, that men may see in it their mirror and their pattern. And the visible world is as it were one great holy book, full of pictures, and parables, and analogies, and other instructive things. All the things we see have their own beautiful and deep meanings."

"The beautiful white clouds, the rosy sunset, the storm-winds, the soft breezes laden with the fragrance of the flowers on a spring morning, the summer sun glowing like a fiery sea, and the silent sparkling of the stars on a winter night, the mighty thunder, and the gentle chirping of the cricket on the hearth; all these things mean more than we can hear with our ears or see with our eyes.

"And the dark mountain forest, and the tall oak trees, the poplars by the stream, the hawthorn hedges, the vineyards and the cornfields, the wild flowers and the clover fields, the friendly violet and the fragrant rose; all these are not there only for our use and pleasure; they are the letters of a wonderful secret writing, written by God Himself, and the thoughts of God lie concealed in them."—*Alban Stolz.*

### Notes and Queries.

**The Editor is always willing to answer in this column, without any fee, all reasonable questions, as soon as possible.**
**But the following rules must be carefully observed:—**
**1. Every communication must be accompanied by the name and address of the writer.**
**2. The question must always bear upon nursing, directly or indirectly.**
**If an answer is required by letter a fee of half-a-crown must be enclosed with the note containing the inquiry.**

*Midwifery.*

(220) Would you kindly let me know if a woman who is not a trained nurse can make a charge for acting as midwife? The doctor is seven miles away, and, as there is no nurse in the village, she does it to oblige her neighbours. I would like to know if she is justified in making a fixed charge?—*Nurse F.*

Certainly, she is entitled to remuneration for her services.

*Home for Aged.*

(221) Can you tell me of a comfortable home for my sister, aged 72? She must not be left alone, and can afford to pay a moderate sum weekly.—*J. N.*

St. Peter's Home and Sisterhood, Mortimer Road, Kilburn, N.W. The Woodside Home, Whetstone, N.

*Bicycle.*

(222) Will anyone sell a lady's bicycle cheap to the nurse of a large scattered country parish?—*Nurse H.*

*Chronic Constipation.*

(223) Can you tell me if there is any cure for a case of constipation which has lasted eight years? 2. Is there any book on the subject?—*E. H. L.*

1. Consult a doctor. 2. "Constipation and its Modern Treatment." By George Herschell, M.D. Price 2s. 6d. Published by H. J. Glaisher.

*Home for Sick Nurse.*

(224) I am a nurse and have been ill for more than two years with dilated heart. I have 10s. a week sick pay from the Royal National Pension Fund for Nurses, but my private resources are now exhausted. Can I find a comfortable home for that sum?—5569.

See reply to J. N. There are others, for which consult "Burdett's Hospitals and Charities." The following might suit:—Lady Smyth's Home, Long Ashton, Bristol, charges 6s. to 10s. a week; St. Cyprian's Home, 31 The Grove, Hammersmith; or, Working Ladies Guild, 251 Brompton Road. You might apply to the Junius Morgan Benevolent Fund for a grant.

*Diphtheria.*

(225) Is there any convalescent home where a nurse recovering from diphtheria could be received for a small charge. Near London, or on the East Coast preferred.—*J. B.*

You are more likely to get what you want by private advertisement than by any other means. Buo unless you have completely recoverd from the infection, we do not see how you are to travel there without breaking the law.

*Maternity Fee.*

(226) Can you tell me if it is usual for a maternity nurse to charge full fees from the date of her engagement, even if her services are not required until a fortnight later?—*Nurse.*

Yes, unless other arrangements have been made previously.

*Address.*

(227) I see in your report "Progress in Cancer" that Mr. Marmaduke Shield gives important points. I have a patient, who, if it were possible, would like to consult him. Can you give me his address?—*District Nurse.*

Refer to the Medical Directory.

*San Francisco.*

(228) I am going to San Francisco, and would be glad of any information as to private nursing there.—*Nurse.*

Write to the Superintendent of Nurses, the Hospital for Children and Training School for Nurses, 3760 California Street, San Francisco.

*Scarlet Fever.*

(229) Can the matron of a nurses' home claim compensation from the Urban Council for the loss of a nurse's services who has contracted scarlet fever whilst nursing at the Isolation Hospital, the matron, of course, paying the nurses full salary all the while?—*E. M. L.*

No.

*Midwifery.*

(230) How far may an untrained woman act as midwife? Has a fatal mistake to be made before she can be restrained?—*Conservative.*

If a midwife does her best, and makes no pretence of special knowledge, she cannot be restrained. She may, of course, be punished for neglect when that can be proved.

**Standard Books of Reference.**

"The Nursing Profession: How and Where to Train." 2s. net; post free 2s. 4d.
"Burdett's Official Nursing Directory." 3s. net.; post free, 3s. 4d.
"Burdett's Hospitals and Charities." 5s.
"The Nurses' Dictionary of Medical Terms." 2s.
"Burdett's Series of Nursing Text-Books." 1s. each.
"A Handbook for Nurses." (Illustrated.) 5s.
"Nursing: Its Theory and Practice." New Edition. 3s. 6d.
"Helps in Sickness and to Health." Fifteenth Thousand. 5s.
"The Physiological Feeding of Infants." 1s.
"The Physiological Nursery Chart." 1s.; post free, 1s. 3d.
"Hospital Expenditure: The Commissariat." 2s. 6d.

All these are published by THE SCIENTIFIC PRESS, Ltd., and may be obtained through any bookseller or direct from the publishers, 28 and 29 Southampton Street, London, W.C.

### Travel Notes.

By Our Travelling Correspondent.

### LXXXI.—IN THE WEST COUNTRY.

Circumstances have brought me into a very old-world corner of the west country, and though it is holiday time, I shall be magnanimous and tell you something of the beauties and pleasures of Somersetshire. I am established in a small village near to Glastonbury and not far from Wells, whilst Bridgewater and the historic field of Sedgemoor are within driving or cycling distance, or even for very modest pedestrians.

### The Journey from London.

The third-class ticket to Ashcott, which is our nearest station, is 11s., certainly not ruinous, and I can give the address of a lady who receives paying guests on very moderate terms to anyone who seriously wishes to come here. As one comes further and further west, the stations become more] quiet and sleepy. There are one or two changes, always at Templecombe and sometimes at Evercreech Junction, though at others a word to the guard at Templecombe ensures the passenger being duly deposited at Ashcott. Meare, the village in which I am staying, is about a mile and a half from the station along a level road, with peat moors each side.

### The Village Itself.

As you may suppose, its attractions are not numerous, but its position as a cycling centre is delightful, and the roads are excellent. Somersetshire is not a hilly county on the whole, and we lie between the Mendips and the Quantocks. The church is very ancient, but I cannot say it is very interesting, its chief architectural point being one of those curious slanting openings between the nave and the sanctuary called, I believe, a "squint," because they were made so that those sitting at an unpropitious angle might be able to see the altar. The walls and pillars have been scraped and cleaned with cruel ardour, and some atrocious ash pews substituted for the old oak.

### The Manor Farm.

Close to the east end of the church stands an old farm, once the manor farm attached to the Abbey of Glastonbury, and used by the abbots as a kind of home of rest when they wanted a little immunity from the cares of monastic government. In this farm there is a splendid room now used for wool and cheese, where, history says, Robert Whiteing the last abbot was sitting when seized by Henry VIII.'s commissioners on a trumped-up charge of treason. Wolsey had his eye on the rich Abbey lands and justice was unknown when he desired his own or his master's aggrandisement. In the poultry-yard at the back is the disused entrance to a subterranean passage, supposed to lead to Glastonbury Abbey, some three and a half miles distant. It has been traced some distance in that direction, and as there is also a similar entrance within the precincts of the Abbey, the tradition is probably quite correct.

### The Ruins and Town of Glastonbury.

In early days Glastonbury lay in a kind of marsh in which there were several islands. This is the district spoken of as Avalon. You remember Tennyson makes King Arthur speak of "The Island Valley of Avilion," where he went to "heal me of my grievous wound." He died there, and was buried in the Abbey church. Later, Queen Guinevere was buried by his side, and it is supposed that their bones still rest there, though now unmarked. The marshy nature of the land made fish plentiful, and tithes were taken in fish as well as in grain. In Meare a very ancient building still stands called the Fish House, where these tithes were once officially received.

### The Abbot's Barn and Kitchen.

These are at some distance from each other; the kitchen is attached to the Abbey buildings, but the barn is half a mile away. In the latter, enormous quantities of grain, hay, etc. . . . could be stored. It is a vast structure and in almost perfect condition, though erected in or near the year 1420. It is cruciform, and has two very large and handsome doorways opposite each other. There are no windows, but free ventilation is secured by several slits and some trefoil openings. Of the Abbey buildings, the only one not in ruins is the abbot's kitchen, and most remarkable it is. The form is really square, but the effect is octagonal, because the four corners are cut off by the placing of an enormous fireplace in each. In one only is there an oven. The roof meets almost in a point attached to a double lantern. This roof interiorly is supported by eight gracefully curved ribs, leaving holes round the lantern for ventilation and the escape of fumes from below. Outside there are some almost unique circular buttresses.

### St. Joseph's Chapel.

There is more left of this chapel than of the bigger abbey, and enough remains to show how exquisite was the work. St. Joseph of Arimathea, so identified with Glastonbury, is supposed to have planted his staff on Tor Hill, where it budded and blossomed and became the Holy Thorn. To this day there is a kind of thorn in Glastonbury which blooms twice every year, in May and at Christmas, and which will not flourish in any other district.

In 1539 Henry VIII. confiscated the Abbey, its lands and emoluments, and the venerable abbot was tried for high treason in the vast hall of the Bishop's Palace at Wells, from whence he was dragged on a hurdle to Tor Hill, there hung with his prior and sub-prior, and whilst still living their bodies were taken down drawn and quartered as was the savage custom in what we are addicted to calling the "good old days!"

It is not surprising that so little of the buildings remain, for I was told not only that the more modern houses of Glastonbury are built with the old stone, but that even the roads round for some miles have been constructed with the same materials!

---

### TRAVEL NOTES AND QUERIES.

Rules in Regard to Correspondence for this Section.—All questioners must use a pseudonym for publication, but the communication must also bear the writer's own name and address as well, which will be regarded as confidential. All such communications to be addressed "Travel Editor, 'Nursing Mirror,' 28 Southampton Street, Strand." No charge will be made for inserting and answering questions in the inquiry column, and all will be answered in rotation as space permits. If an answer by letter is required, a stamped and addressed envelope must be enclosed, together with 2s. 6d., which fee will be devoted to the objects of the "Hospital" Convalescent Fund. Any inquiries reaching the office after Monday cannot be answered in "The Mirror" of the current week.

North Italy for Three Months (Artist).—You would find the following route suitable. Touch at Milan to see the Cathedral, but do not linger. Spend a week at Verona, from which visit Mantua and Cremona, then go on to Venice, where if possible spend three weeks. Then to Bologna for a week or ten days, visiting Modena and Ferrara and Ravenna if you can, then down to Florence, spending one or two nights at Pistoja en route. A month should be spent at Florence, from which you must see Vallombrosa. I think you would enjoy three days there. Prato can be seen in a day from Florence—Fiesole the same. A day and night at Pisa, a week at Lerici. This place is close to Spezia, but Spezia itself is now somewhat spoilt, then home by Genoa.

Holland or Belgium (Wyeside).—Both countries lie low, so must be a little damp late in the season. Your terms are really too low; I cannot hold out hopes of anything at less than 6 fcs., which is about £1 13s. 6d. a week each, and then there will be tips; it is not possible to get good accommodation at this season at inclusive rates of £1 10s. per week. Either Brussels or Ghent would be the best centres; from either you can visit almost all the chief Belgian cities in a day, and travelling is very cheap. Ghent is cheaper than Brussels, but of course not so amusing and interesting. I think you might go to the Hôtel de la Paix or the Hôtel Aux Arms de Zeelande for 6 fcs. or 6.50. If you stay at Brussels go to Hôtel du Rhin, 14 Rue de Brabant, or Hôtel de Barière. Take two woollen dresses, one thick and one thin, and a few blouses of different thicknesses. It will probably be cold. You pay for your luggage, so there is no limit; but for all reasons the less the better. If you would like a week in the Ardennes go to Dinant on the Meuse, it is lovely scenery, Hôtel des Familles from 7 fcs.

